# Precision high-speed quantum logic with holes on a natural silicon foundry platform

**DOI:** 10.1038/s41467-026-76231-x

**Published:** 2026-08-27

**Authors:** Isaac Vorreiter, Jonathan Y. Huang, Scott D. Liles, Joe Hillier, Ruoyu Li, Bart Raes, Stefan Kubicek, Julien Jussot, Sofie Beyne, Clement Godfrin, Sugandha Sharma, Danny Wan, Nard Dumoulin Stuyck, Will Gilbert, Chih Hwan Yang, Andrew S. Dzurak, Kristiaan De Greve, Alexander R. Hamilton

**Affiliations:** 1 School of Physics, University of New South Wales, Sydney, NSW 2052 Australia; 2 IMEC, Leuven, Belgium; 3 School of Electrical Engineering and Telecommunications, University of New South Wales, Sydney, NSW 2052 Australia; 4 Diraq Pty. Ltd., Sydney, NSW Australia; 5 Department of Electrical Engineering (ESAT), KU Leuven, Leuven, Belgium; 6 Present Address: Applied Materials, Santa Clara, CA USA

**Keywords:** Quantum information, Electrical and electronic engineering, Qubits

## Abstract

Silicon spin qubits in gate-defined quantum dots leverage established semiconductor infrastructure and offer a scalable path toward transformative quantum technologies. Holes spins in silicon offer compact all-electrical control, whilst retaining the salient features of a quantum dot qubit architecture. However, silicon hole spin qubits are not as advanced as electrons, due to increased susceptibility to disorder and more complex spin physics. Here we demonstrate single-qubit gate fidelities up to 99.8% and a two-qubit gate quality factor of 240, indicating a physical fidelity limit of 99.7%. These results represent the highest performance reported in natural silicon to date, made possible by fast qubit control, exchange pulsing, and industrial-grade fabrication. Notably, we achieve these results in a near-identical device as used for highly reproducible, high-fidelity electron spin qubits. With isotopic purification and further device-level optimisations, silicon hole spin qubits are poised to unlock a new operation regime for quantum CMOS architectures.

## Abstract (web summary)

Silicon hole spin qubits promise fast electrical control but have lagged behind electron spins due to disorder sensitivity and complex spin physics. Here, the authors demonstrate record single-qubit gate fidelities and two-qubit gates with high quality factor in natural silicon using an industrial CMOS process.

## Introduction

Among the diverse approaches to quantum computing, spin qubits in silicon are increasingly recognised as a competitive platform due to their compatibility with industrial semiconductor technology and potential for large-scale integration^1–5^. Hole spin qubits, which use the *j*_*z*_ = ± 3/2 ground state to encode the $\left\vert 0\right\rangle$ and $\left\vert 1\right\rangle$ qubit states, stand out due to their strong spin-orbit interaction (SOI), which unlocks ultrafast spin control via electric-dipole spin resonance (EDSR)^6–11^ and facilitates spin-photon coupling^12^. While the hyperfine noise for silicon hole spins is expected to be similar to that of electrons^13^, the presence of strong spin-orbit interaction may allow the hyperfine coupling to be tuned electrically and magnetically ^14–17^. Although holes confined in quantum dots have been realised in several different silicon MOS architectures^10,18,19^, the simultaneous fulfilment of all core DiVincenzo criteria^20,21^ has not yet been demonstrated.

Utilising a *p*-type device fabricated in a modern, cutting-edge 300 mm CMOS pilot line^22^, we implement all essential components required for scaling up hole spin qubits – single-shot readout, deterministic initialisation, and one- and two-qubit logic. We report high-fidelity one- and two-qubit gates in natural silicon with 4.7% ^29^Si spinful nuclei, without the need to isotopically purify the silicon substrate. We find the quality factors of single- and two-qubit gates corresponding to physical fidelity limits well above the surface code threshold^23^. We measure single-qubit fidelities that represent the highest level in natural silicon MOS quantum dots and perform an initial study on the single-qubit control and coherence properties. These results are obtained in devices with near-identical geometries and fabrication procedures used in recent high-fidelity electron spin qubit measurements^24^ (the main difference being the peripheral *p*-type contacts). This platform therefore enables the possibility of quantum CMOS chips that combine both electrons and holes, to take advantage of excellent coherence properties with high-speed control.

## Results

### Device operation and readout

**Fig. 1:**
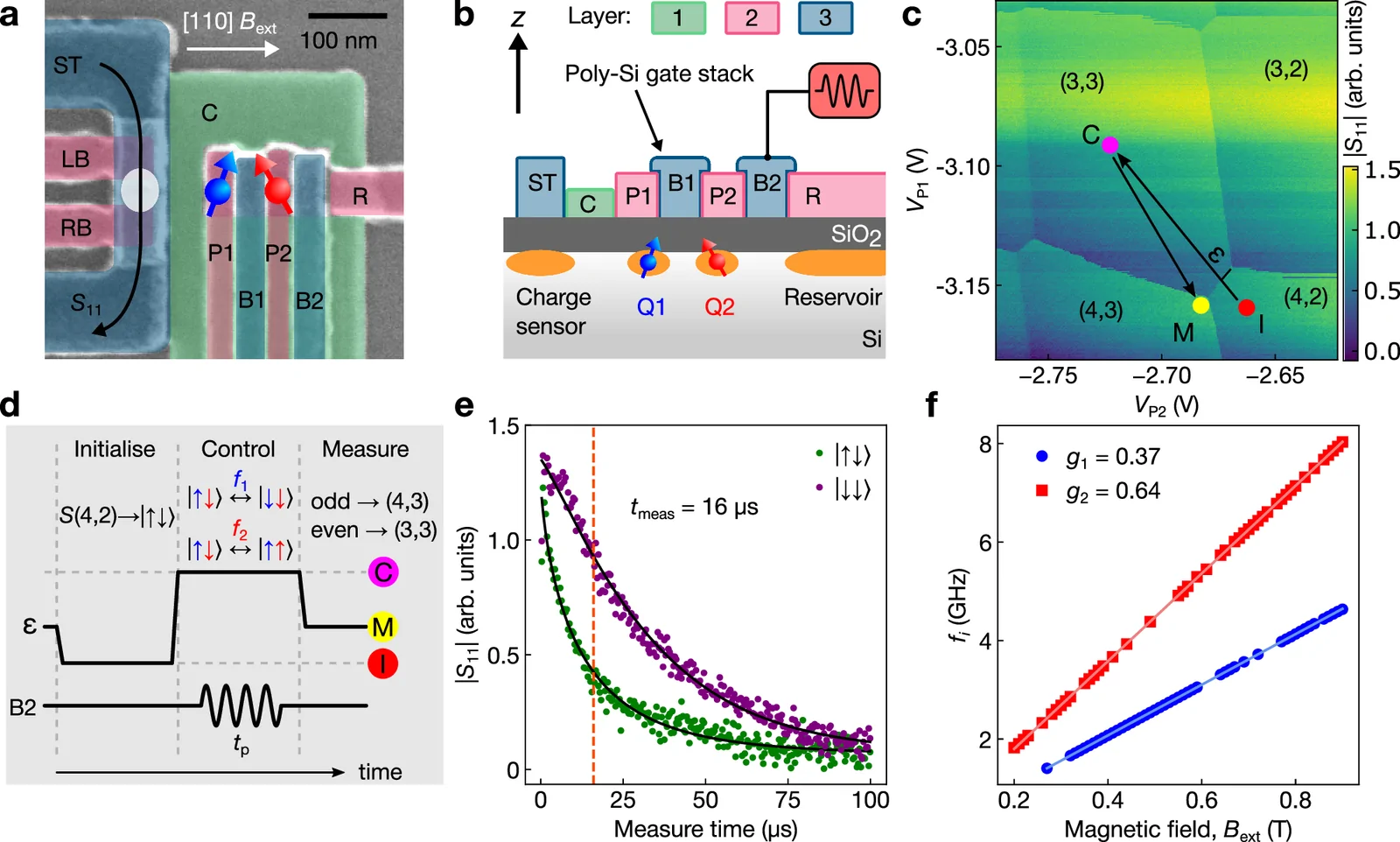
Operation and readout of a hole-based two-qubit unit cell in natural silicon MOS. **a** False-coloured scanning electron microscope (SEM) image and **b** schematic of the device. Qubits Q1 and Q2 are formed in quantum dots defined by the C, B1, and B2 gates, and the hole occupation is controlled using the P1 and P2 gates. Qubit control is performed using a microwave tone on B2, and single shot spin-to-charge readout is performed with radio frequency reflectometry on an adjacent sensor dot. **c** Stability diagram at the (3,3)-(4,2) region where the qubits are operated, equivalent to a (1,1)-(2,0) transition. Single shot readout is performed via latched Pauli Spin Blockade. **d** Pulse scheme for single-spin manipulation, consisting of spin initialisation (I), control (C) and measure (M) steps. In the control step an *X*(*θ*) gate is created by applying a microwave pulse resonantly to one of the qubits to rotate the spin by angle *θ*. **e** Charge sensor signal following the measurement scheme in (d) as a function of measure time for states in the $\left\vert \uparrow \downarrow \right\rangle$ and $\left\vert \downarrow \downarrow \right\rangle$ states. Readout fidelity is maximised when *t*_meas_ = 16 *μ*s. **f** Individual qubit addressability: qubit Larmor frequencies *f*_*i*_ as a function of magnetic field *B*_ext_. Linear fits to *f*_*i*_ = *g*_*i*_*μ*_B_*B*_ext_/*h* yield *g*-factors of *g*_1_ = 0.37 and *g*_2_ = 0.64. All measurements performed in a dilution refrigerator with a base temperature of 10 mK.

Figure 1a shows a SEM of the device, and Fig. 1b shows a corresponding schematic of the device cross-section. The device is fabricated on a 10 nm SiO_2_ oxide on an undoped natural silicon substrate. The gates consist of a 3-layer polysilicon stack fabricated using electron beam lithography and subtractive patterning^25^.

The two quantum dots are defined by the plunger gates P1 and P2, which control the dot occupation. The dots are laterally confined by the C gate and the barrier gates B1 and B2, and their charge occupations are controlled by the plunger gates P1 and P2. A 2D reservoir of holes is formed under the R gate. The hole occupancy in each dot is measured with an adjacent single-hole transistor (SHT) integrated with radio-frequency (RF) reflectometry^26^, which provides a high readout bandwidth of  ~ 2 MHz.

To configure our hole quantum dots as spin qubits, we tune the double dot system into the weakly-coupled regime, where the tunnel coupling is much less than the thermal energy of the reservoir *t*_c_ ≪ *k*_B_*T*. We control the number of holes in each dot with P1 and P2, monitored with the charge sensor, as shown in Fig. 1c. The periodic loading of each of the two dots, and the interdot coupling, lead to the characteristic ‘honeycomb’ structure of a double-dot charge stability diagram. In this way, we track the dot occupation down to the last hole in each dot, demonstrating full control over the absolute number of holes in each dot (Supplementary Fig. 3). Knowing the occupation is important, as it sets the nature of the ground state, which strongly influences the properties of the qubit.

Numerical calculations of the electrostatic potential in similar planar MOS devices show that the holes are strongly confined against the heterointerface, into a ‘pancake’-like quantum dot with characteristic length scales of  ~ 10 nm in the z-plane and  ~ 50 nm in the xy-plane^27^. This confinement lifts the degeneracy of the light and heavy hole bands, with only heavy hole states occupied, and the light hole states  ~ 10 meV higher in energy^28^. The in-plane effective mass of the holes is approximately 0.6*m*_0_ - 0.7*m*_0_^29^, and the ground state is expected to be primarily heavy-hole-like^27^. Ultimately, the LH-HH splitting will strongly influence the qubit gate operation speed and decoherence times.

To operate the spins as qubits, we need the ability to initialise and measure the spin states. Figure 1c shows the stability diagram in the (3,3)-(4,2) regime, where (*N*_1_, *N*_2_) denotes the hole occupation of the dots under P1 and P2, respectively. These hole occupations are chosen to allow readout via Pauli Spin Blockade (PSB)^30–32^, since spin-wise, they map to an equivalent (1,1)-(2,0) regime^33^. A single shot measurement is performed using the pulse sequence shown in Fig. 1d, whose trajectory in the voltage space is shown in Fig. 1c. The pulse sequence consists of three steps performing (I)nitialise – (C)ontrol – and (M)easure. Firstly, the spins are initialised at (I) in the (4,2) region as a singlet state, before being pulsed along the P2-P1 detuning axis *ε* into (3,3) where they form a $\left\vert \uparrow \downarrow \right\rangle$ state. Secondly, single-qubit control is performed within the (3,3) region at (C) using microwave pulses. Lastly, a projective spin readout is performed at the (M) point using PSB. Additionally, we perform a reference measurement immediately after the pulse sequence to subtract background noise from the result.

In holes, the readout visibility using PSB is reduced in the presence of a large *g*-factor difference Δ*g*^30^. To improve the readout visibility, we augment the spin-to-charge conversion with a latching step, where the singlet and triplet states map to different hole occupations^34,35^. In this regime, the *T*_0_(3, 3) triplet relaxes to the *S*(3, 3) singlet within our readout integration time, the readout distinguishes between even- and odd-parity instead of singlet and triplet states^30,36^. In Fig. 1e, we plot the resulting readout signal versus time to measure the $\left\vert \uparrow \downarrow \right\rangle$ and $\left\vert \downarrow \downarrow \right\rangle$ states, demonstrating that the states can be distinguished. In this work, we use the convention of measuring the probability of the even-parity spin-blocked states *P*_even_. The low visibility observed likely arises from the site-dependent spin anisotropies and short spin relaxation times, as well as non-adiabaticity of the pulse scheme and the long integration time needed for readout. These issues can be overcome with optimisation of the field angle, double latching schemes, and higher bandwidth sensors^37^. Ultimately, using this combination of PSB and charge latching, we can threshold the measurement outcome to perform single-shot readout in *t*_meas_ = 16 *μ*s. Having demonstrated control of the absolute hole number in each dot, as well as fast single-shot readout and qubit initialization, we now move on to coherent qubit control.

### Single-qubit logic

To rotate the individual spins between the $\left\vert \uparrow \right\rangle$ and $\left\vert \downarrow \right\rangle$ states, we exploit the strong SOI to drive spins electrically using a microwave pulse applied to the B2 gate^6^. For silicon holes, it is expected that the dominant driving mechanism is primarily due to spin-orbit EDSR (also known as iso-Zeeman EDSR), with a smaller contribution due to *g*-tensor modulated resonance^7^. When the frequency of the microwave pulse matches the *i*th-qubit’s Larmor frequency *f*_*i*_ = *g*_*i*_*μ*_B_*B*_ext_/*h*, the spin is driven coherently via EDSR. In Fig. 1f, we plot the measured Larmor frequency as a function of magnetic field *B*_ext_. From this, we find the *g*-factors to be *g*_1_ = 0.37 and *g*_2_ = 0.64 for Q1 and Q2, respectively. We account for this difference in *g*-factor from the observed effective DC Stark shift resulting from the electrical tuneability of the *g*-factor (d*g*/d*V* ≈ 0.7V^−1^) and the  ~ 0.45 V difference in plunger gate voltages.

**Fig. 2:**
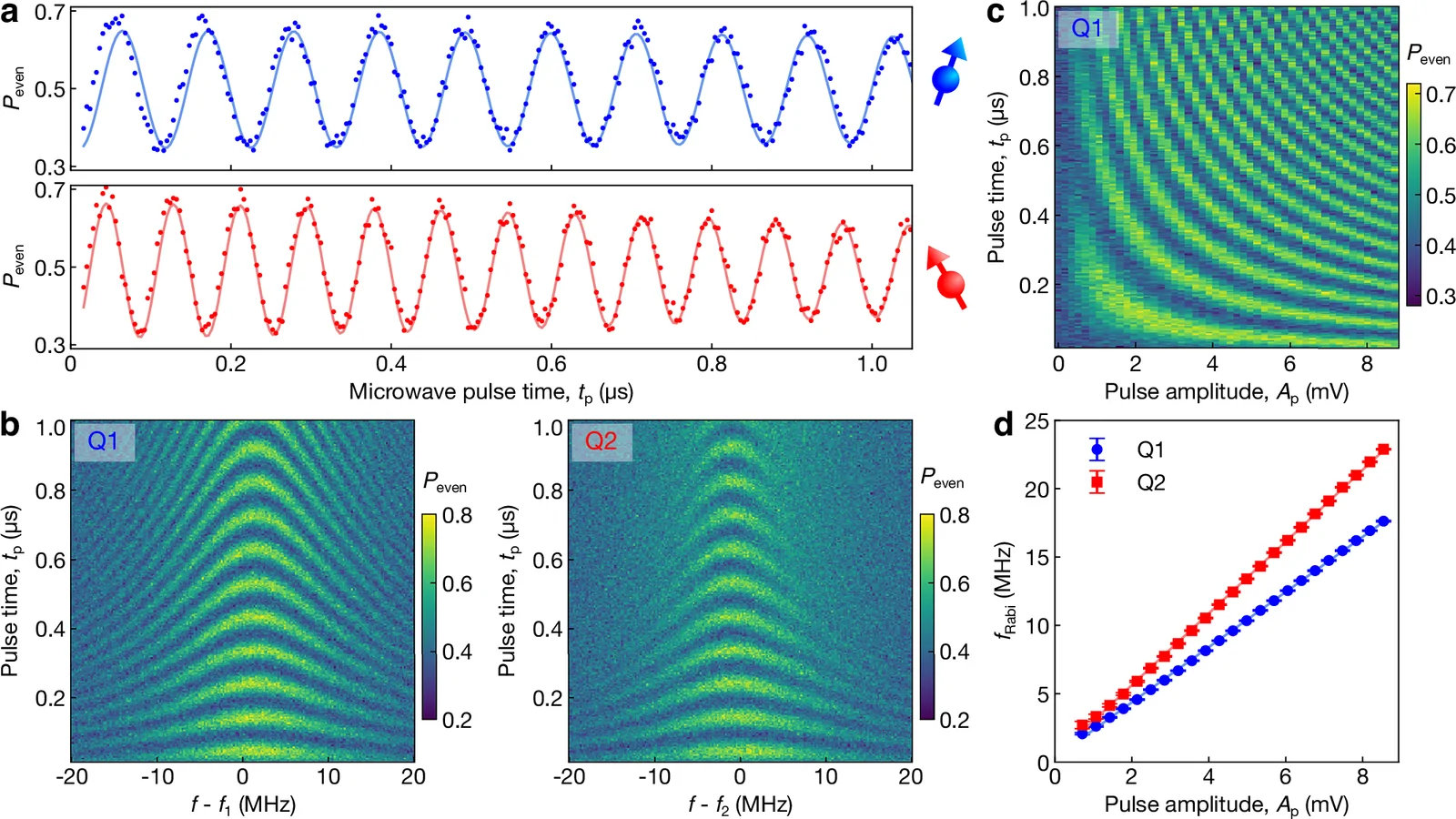
Fast electrically driven single-qubit control for Q1 and Q2. **a** Spin state determined from the spin blocked probability as a function of resonantly-driven microwave pulse time for Q1 (blue) and Q2 (red). The solid lines are fits to a decaying sinusoid given by $P({t}_{{{\rm{p}}}})={c}_{1}\cos (2\pi {f}_{{{\rm{Rabi}}}}{t}_{{{\rm{p}}}}+\phi )\exp (-{({t}_{{{\rm{p}}}}/{T}_{2}^{{{\rm{Rabi}}}})}^{\alpha })+{c}_{2}$. From the fit, we extract ${T}_{2}^{{{\rm{Rabi}}}}$ for each qubit as 3.95 ± 0.13 *μ*s and 1.89 ± 0.07 *μ*s, with errors corresponding to the 95% confidence interval of the fit. **b** Spin state probability as a function of microwave pulse time and frequency detuning, producing a Rabi chevron for each qubit. **c, d** Power dependence of the Rabi oscillations and Rabi frequency *f*_Rabi_, showing the expected linear dependence as a function of microwave pulse amplitude *A*_p_. The Rabi frequencies are extracted using the same fitting function as in (a), with error bars representing the 95% confidence intervals of the fits. All measurements taken at *B*_ext_ = 0.83 T, with *f*_1_ = 4.10 GHz and *f*_2_ = 7.53 GHz.

We investigate the coherent manipulation of the qubit states by driving rotations between the $\left\vert \uparrow \right\rangle$ and $\left\vert \downarrow \right\rangle$ states with EDSR. We demonstrate spin control by varying the length of an on-resonance microwave pulse, *t*_p_, during the (C)ontrol step. The resultant Rabi oscillations are shown in Fig. 2a for both qubits. The frequency and decay of the Rabi oscillations are extracted from a decaying sinusoid fit, yielding Rabi frequencies of 9 MHz and 12 MHz and decay times of 3.95 ± 0.07 *μ*s and 1.89 ± 0.03 *μ*s for Q1 and Q2, respectively. These values can be contextualized for quantum information processing using the gate quality factor ${Q}_{{{\rm{gate}}}}={f}_{{{\rm{Rabi}}}}{T}_{2}^{{{\rm{Rabi}}}}$, which provides an estimate of the number of single-qubit gates that can be coherently executed. In practical quantum information processing applications, more complex pulse schemes are required to implement a universal gate set, thereby introducing additional unitary gate errors and reducing the direct correspondence between fidelity and the gate quality factor. Our results show quality factors of 36 and 23 for Q1 and Q2, which are reasonable in the context of spin qubits^38^. The gate quality factor may be improved by tuning the qubit into a ‘sweet spot’ using magnetic field direction and gate voltage^17,39–41^.

In Fig. 2b, we extend the Rabi experiment by varying the pulse length for different frequency detuning Δ*f*_*i*_ = *f* − *f*_*i*_ of the microwave pulse. This produces a Rabi chevron for both qubits. In Fig. 2c, we plot measured Rabi oscillations for different microwave pulse amplitudes *A*_p_. The extracted Rabi frequencies are plotted against *A*_p_ in Fig. 2d, which displays a linear relationship that confirms *f*_Rabi_ ∝ *A*_p_. This leads to a maximum *f*_Rabi_ = 23 MHz, or equivalently, a minimum gate time of *t*_*π*/2_ = 10 ns. In our experiment, the speed is limited by attenuation in the coaxial lines and by the maximum amplitude we can apply with our microwave source, preventing us from observing a non-linear driving regime. Extrapolating from the linear slope in Fig. 2d, we predict that the maximum amplitude we could apply to the qubit under these bias conditions is limited by the charging energy voltage *V*_*C*_ ~ 100 mV, leading to a bound on the Rabi frequency of  ~ 200 MHz. In terms of driving speed, the measured hole spin qubits exceed those of electrons in near-identical devices^24^ by a factor of  ≈ 20. In structurally similar electron SiMOS devices, gate quality factors of up to 100 have been reported ^42^.

### Two-qubit logic

**Fig. 3:**
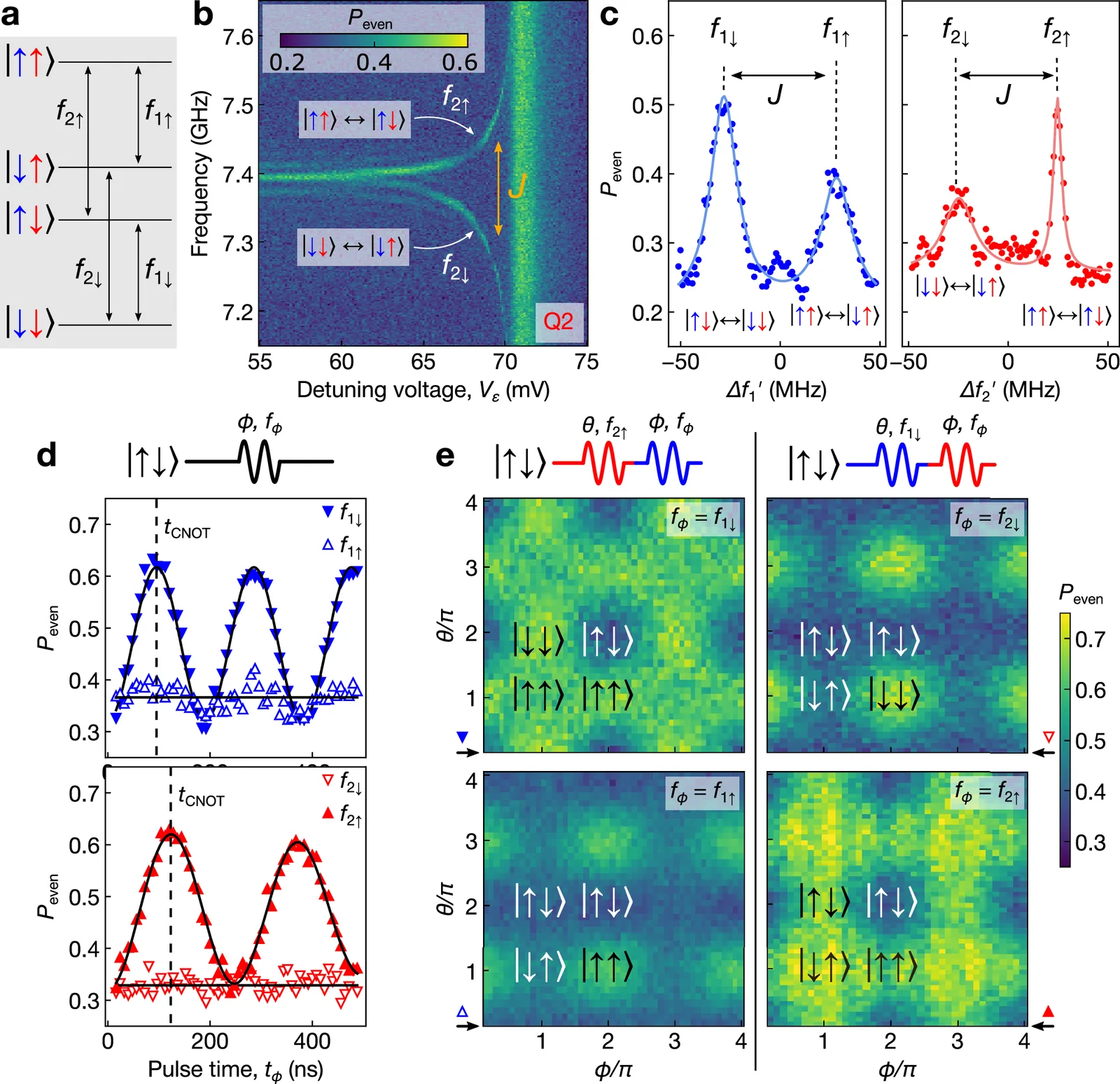
Exchange interaction and controlled rotation (CROT) operation. **a** Energy level diagram of the four non-degenerate spin states in the presence of finite exchange *J*. **b** EDSR spectrum as a function of P2-P1 detuning voltage *V*_*ε*_. As detuning *ε* approaches the anticrossing (*V*_*ε*_ → 72 mV), the exchange interaction splits the single qubit frequency into separate branches corresponding to the $\left\vert \uparrow \downarrow \right\rangle \leftrightarrow \left\vert \uparrow \uparrow \right\rangle$ and $\left\vert \downarrow \uparrow \right\rangle \leftrightarrow \left\vert \downarrow \downarrow \right\rangle$ transitions. **c** EDSR spectrum vs frequency detuning taken at *V*_*ε*_ = 66 mV demonstrating an exchange splitting of *J* = 53 ± 4 MHz for both qubits. Fits to the data are made using a bimodal Lorentzian. **d** CROT: *P*_even_ as a function of the qubit pulse time *t*_*ϕ*_ on Q1, demonstrating that the rotation of one qubit is conditional on the spin state of the other. The lower panel is the same, but for Q2. **e** Full CROT control diagram: Same as for (d), but with an additional pulse of length *t*_*θ*_ and frequency *f*_1*↓*_ (left panels) or *f*_2*↑*_ (right panels). This demonstrates two-qubit logic.

A common approach to implementing two-qubit logic with spins is through the exchange coupling *J* of a double quantum dot^20,43^. *J* depends strongly on the wavefunction overlap between spins, meaning that *J* increases rapidly as the two spins are brought closer, either by pulsing *V*_*ε*_ near the (3,3)-(4,2) anticrossing, or by biasing B1 to be more accumulating. The increase in *J* results in a splitting between the individual qubit resonant frequencies, as shown in Fig. 3a. This indicates that the *i*th-qubit can only be addressed at *f*_*i*,*σ*_, which depends on the spin state *σ* of the other qubit.

To measure the exchange splitting, we first initialise in the $\left\vert \uparrow \downarrow \right\rangle$ state, which is not an eigenstate of the system near the anticrossing. As *ε* approaches the anticrossing, the qubit resonance is split into two, given by *J*. This corresponds to transitions between the $\left\vert \uparrow \downarrow \right\rangle \leftrightarrow \left\vert \uparrow \uparrow \right\rangle$ and $\left\vert \downarrow \uparrow \right\rangle \leftrightarrow \left\vert \downarrow \downarrow \right\rangle$ states, as shown for Q2 in Fig. 3b, where we plot the EDSR spectrum versus *V*_*ε*_. Here we observe that *J* gets larger as *V*_*ε*_ → 72 mV, corresponding to the (3,3)-(4,2) anticrossing. We can extend this experiment by comparing the EDSR spectrum at (*V*_*ε*_ = 66 mV) as shown in Fig. 3c. This clearly demonstrates that both qubit resonance frequencies are split by *J* = 53 ± 4 MHz.

We explore two implementations of two-qubit logic gates – the driven controlled rotation (CROT) gate^9,19,44–46^ and the decoupled controlled-phase (DCZ) gate^47–51^. To enact the CROT, we initialise into the $\left\vert \uparrow \downarrow \right\rangle$ state and apply a microwave pulse of length *t*_*ϕ*_ at one of the four exchange-split resonance frequencies *f*_*i*,*σ*_, followed by a readout operation. The resulting spin-blocked probability is shown in Fig. 3d. In the case where we drive on either *f*_1*↓*_ or *f*_2*↑*_, we see oscillations in the even-parity probability. However, if we drive at *f*_1*↑*_ or *f*_2*↓*_, the qubits remain in a spin-unblocked state. This demonstrates rotations on each qubit, conditional on the state of the other qubit. In our experiment, a CROT-*π* rotation can be performed in *t*_CROT_ = 100 ns (125 ns) for Q1 (Q2). Together with the extracted ${T}_{2}^{\,{\rm{CROT}}}$ times (Supplementary Fig. 6), we calculate quality factors ranging between 13 and 38.

In the driven CROT scheme, the choice of which of the four *f*_*i*,*σ*_ frequencies to use is arbitrary, since a two-qubit gate set only requires one entangling gate. Hence, we can confirm that all four frequencies indeed allow for CROT. To achieve this, we extend on the initial experiment by first applying a pulse on the control qubit of length *t*_*θ*_. This control pulse is at *f*_1*↓*_ if the target is Q2 (left column), or *f*_2*↑*_ if the target is Q1 (right column). The resulting oscillation ‘checkerboard’ maps are shown in Fig. 3e. For *t*_*θ*_ = 0, the horizontal linecuts of each sub-panel exactly match the plots in Fig. 3d as indicated by the arrow at the base of each sub-panel. When the length of the first pulse is timed to give an odd number of spin rotations (*θ* = *nπ*), we observe an ‘inversion’ of the control operation: oscillations now occur for *f*_1*↑*_ (bottom-left panel), whereas no oscillations occur for *f*_1*↓*_ (top-left panel) as they did for *θ* = 0. Similarly, oscillations now occur for *f*_2*↓*_ (top-right panel), whereas no oscillations occur for *f*_2*↑*_ (bottom-right panel). The control operation is then ‘restored’ when *θ* = 2*nπ*. This set of measurements demonstrates control of one qubit state conditional on another qubit state, and the canonical controlled-NOT (CNOT) gate can then be realised by combining the CROT-*π* gate with a virtual $\sqrt{{{\rm{Z}}}}$-gate^45^.

**Fig. 4:**
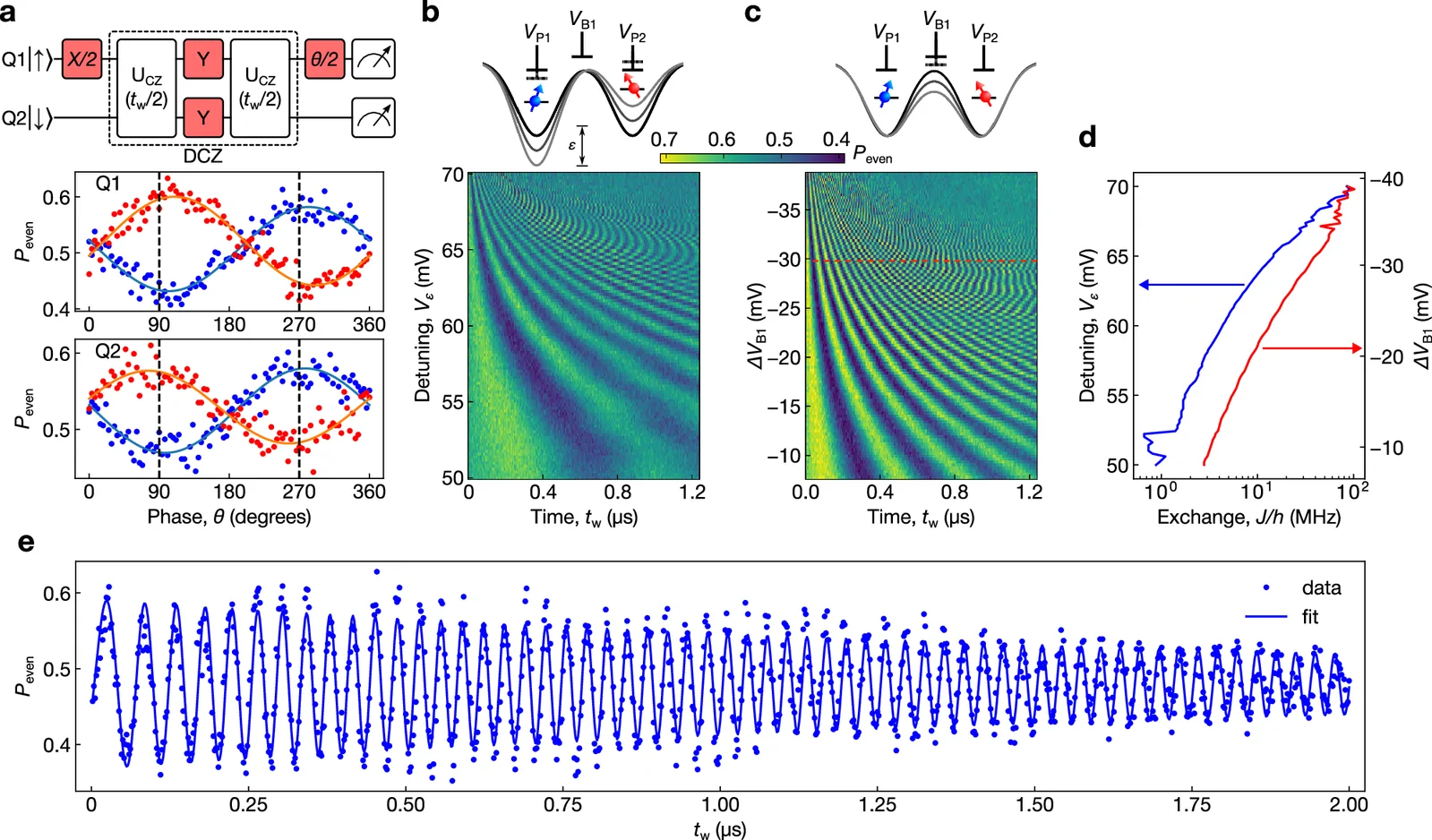
Decoupled controlled phase (DCZ) operation. **a** Quantum circuit diagram (top) used to perform the Ramsey-like calibration of the DCZ gate, with single-qubit driven operations indicated in red. Measured even-parity probability (middle, bottom) of the Ramsey-like sequence above as a function of the phase of the second *π*/2 pulse, *θ*. For each measurement, we initialize the control qubit in its fiducial state (blue curve) or in its spin-flipped state using a *π*-pulse (red curve). Each measurement is performed with exchange *J*/*h* = 20 MHz. **b** Measured DCZ oscillations as a function of the CZ operation time *t*_*w*_ and the detuning pulse depth *V*_*ε*_. **c** Same as for (b), but with the exchange now controlled primarily by the interdot barrier gate B1. **d** Exchange *J* extracted from the fast Fourier transforms of the DCZ oscillations in (**b**) and (**c**). Here we show that *J* is tunable over approximately 2 decades. **e** Linecut of DCZ exchange maps taken at Δ*V*_B1_ = − 29.6 mV. From the fit, we extract a two-qubit gate quality factor of 240, corresponding to an upper limit of the fidelity of 99.7%.

A simpler but faster implementation of two-qubit gates is the CZ family. When exchange is switched on, a qubit accumulates phase at different rates conditional on the state of the other qubit, with the difference given by *J*. We enable the exchange interaction using square pulses with a rise time of  ~ 1 ns. In the presence of an effective DC Stark shift from the electrically tuneable *g*-factor, the CZ operation will accumulate an unconditional phase rotation given by the Zeeman energy difference between the two levels of the exchange pulse^47^, which for this device is on the order of  ~ 100 MHz for Q1 and  ~ 10 MHz for Q2. To cancel out this unwanted phase rotations on individual qubits and extend coherence, we incorporate a decoupling pulse to create DCZ operation, as illustrated in Fig. 4a. As a result of this pulse sequence, given a certain *J* level, the phase of the target qubit oscillates at the same frequency with opposite phases when the control qubit is $\left\vert \uparrow \right\rangle$ and $\left\vert \downarrow \right\rangle$. A DCZ-*π* operation can be implemented by calibrating the exchange wait time *t*_w_ such that the target qubit accumulates a phase of $\frac{\pi }{2}$ when the control qubit is $\left\vert \uparrow \right\rangle$ and $-\frac{\pi }{2}$ when the control qubit is $\left\vert \downarrow \right\rangle$. The canonical DCZ gate can then be formed by applying an unconditional phase correction of $\frac{\pi }{2}$ to both qubits after the exchange-echo-exchange process.

Figure 4b, c show DCZ oscillations as a function of *V*_*ε*_ and the variation in B1 gate voltage *V*_B1_, respectively. As a result of the fast oscillations and the decoupling pulse, we acquire substantially increased quality factors. An example time trace is shown in Fig. 4d, where *Q*_gate_ = 240 is observed. These quality factors, obtained from natural silicon, surpass those from the best academic-lab-built devices in isotopically purified silicon (from 800 ppm to 50 ppm)^50,52^ and approach the level in state-of-the-art foundry devices with 400 ppm  ^29^Si^24^.

### Coherence times and fidelities

**Fig. 5:**
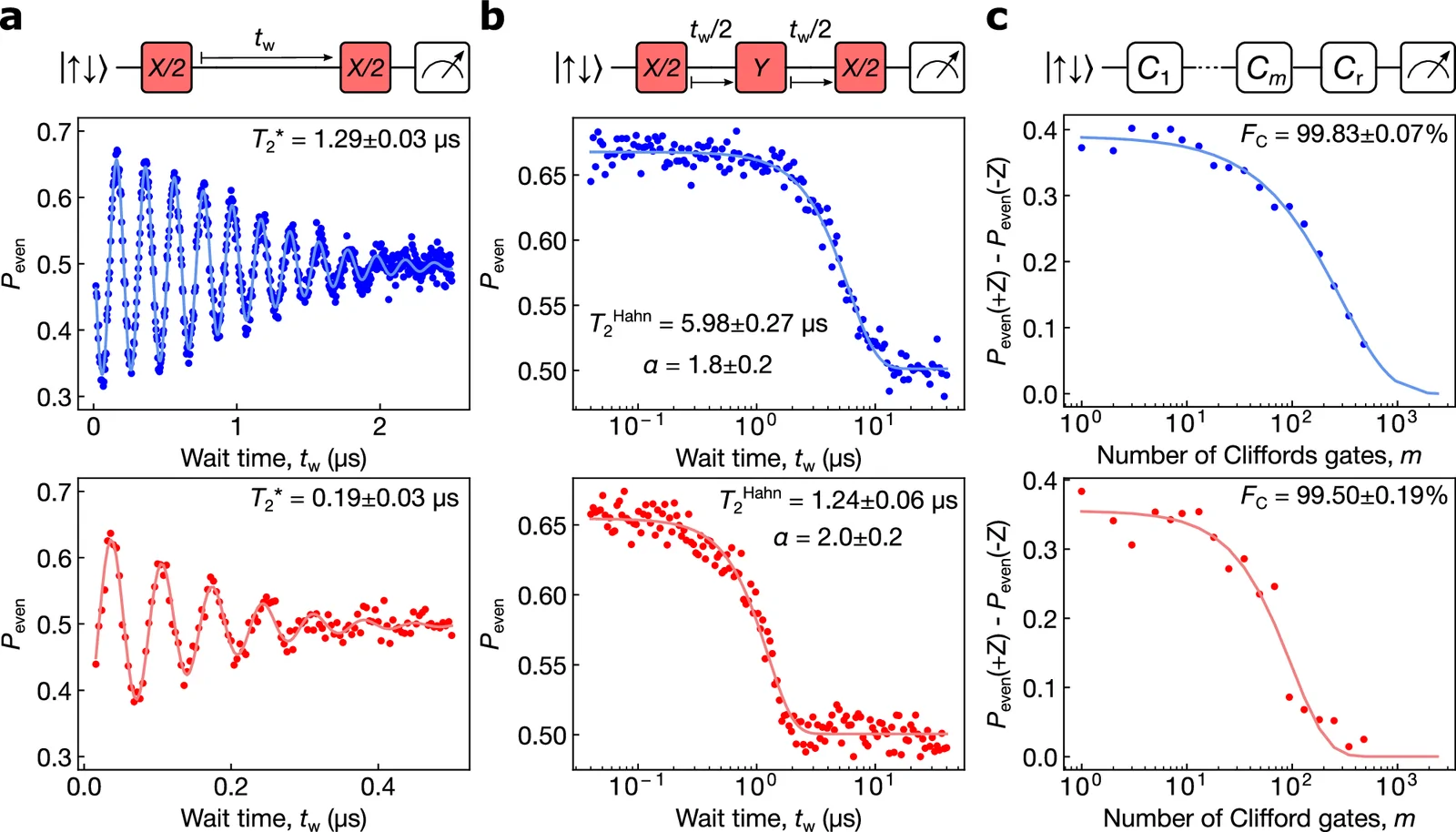
Qubit performance. **a** Free-induction decay (Ramsey sequence), showing the resulting spin blocked probability as a function of wait time *t*_w_. The solid lines are fits to a decaying sinusoid ${c}_{1}\cos (2\pi \Delta f{t}_{{{\rm{w}}}}+\phi )\exp (-{({t}_{{{\rm{w}}}}/{T}_{2}^{*})}^{\alpha })+{c}_{2}$, from which we extract ${T}_{2}^{*}=1.29\pm 0.03\,\mu {{\rm{s}}}$ and 0.19 ± 0.03 *μ*s for Q1 and Q2. **b** Spin refocusing with a Hahn echo. By interleaving a *Y*(*π*) gate in the Ramsey sequence, we refocus the spin dephasing and thereby extend the spin coherence time. Solid lines show fits to ${c}_{1}\exp (-{({t}_{{{\rm{w}}}}/{T}_{2}^{{{\rm{Hahn}}}})}^{\alpha })+{c}_{2}$, yielding ${T}_{2}^{{{\rm{Hahn}}}}=5.98\pm 0.27\,\mu {{\rm{s}}}$ and 1.24 ± 0.06 *μ*s. **c** Single-qubit Clifford sequence for randomised benchmarking, with *m* the number of Clifford gates. The decay of the sequence is fitted to *c*_1_*f*^*m*^ + *c*_2_, from we extract a Clifford fidelities *F*_C_ = (1 + *f*)/2 of 99.83 ± 0.07% and 99.50 ± 0.19% for Q1 and Q2. All experiments performed at *B*_ext_ = 0.83 T. The errors correspond to the 95% confidence interval of the fitting.

Qubit coherence is characterised by the ${T}_{2}^{*}$, ${T}_{2}^{{{\rm{Hahn}}}}$, and ${T}_{2}^{{{\rm{CPMG}}}}$ times, which reflect the dephasing from low, intermediate and high frequency noise sources. We evaluate the ${T}_{2}^{*}$ time through a Ramsey free-induction decay experiment. Here, we apply two X_*π*/2_ pulses with frequency detuning Δ*f*, separated by a wait time *t*_w_. During the wait time, the qubit is exposed to low-frequency longitudinal noise, which causes the qubit to dephase. As shown in Fig. 5a, this measurement produces oscillations with frequency Δ*f* in the spin probability modulated by a decay envelope. From this decay we extract coherence times of ${T}_{2}^{*}=1.24\pm 0.11\,\mu {{\rm{s}}}$ and 0.19 ± 0.02 *μ*s for Q1 and Q2 measured with an integration time of 24 ms per data point. We can decouple the qubit from this low-frequency noise via a Hahn echo sequence, thereby increasing the coherence time of the qubit. This is achieved by interleaving an additional *Y*(*π*) pulse into the Ramsey sequence, and the resulting spin echo is shown in Fig. 5b. Here the spin probability of each qubit decays in characteristic time ${T}_{2}^{{{\rm{Hahn}}}}=5.79\pm 0.38\,\mu {{\rm{s}}}$ and 1.25 ± 0.06 *μ*s for Q1 and Q2, respectively. This confirms that refocussing extends the coherence time of both qubits.

One common source of dephasing for spin qubits is nuclear hyperfine noise. We estimate the dephasing from hyperfine noise by calculating the Overhauser field of the residual ^29^Si nuclei in natural silicon^13^. This yields an estimate of  ≈ 1.5 *μ*s, which is of the same order of magnitude as our measured result for Q1. This finding is consistent with recent results in silicon hole spins^10,17,35^ which indicate that hyperfine noise likely contributes significantly to the total dephasing rate. Furthermore, this dephasing rate is of similar order of magnitude to electrons in natural silicon^53^, but a factor of  ≈ 10 − 100 times lower than for electrons in 400 ppm isotopically enriched silicon^24,54^. Using the same estimation as above, we would expect ${T}_{2}^{*}\approx 10\,\mu {{\rm{s}}}$ for 400 ppm isotopically enriched silicon. This indicates that hyperfine noise may be further suppressed by isotopic purification of the silicon substrate, offering a simple path to significantly improve hole qubit performance.

The second common source of dephasing for spin qubits is charge noise. This is typically present in the form of both 1/f noise and white noise. The ratio between the ${T}_{2}^{*}$ and the ${T}_{2}^{{{\rm{Hahn}}}}$, ${T}_{2}^{{{\rm{CPMG}}}}$ times is a proxy for determining the relative contribution of low-frequency noise to the overall dephasing time. In our study, we see ${T}_{2}^{{{\rm{Hahn}}}}/{T}_{2}^{*}\approx$6 for both qubits. By comparison, for electron spins in 400 ppm silicon, the ratio of ${T}_{2}^{{{\rm{Hahn}}}}/{T}_{2}^{*}$ is  ≈ 10^54^, indicating that high-frequency noise contributes more to hole spins than for electron spins. Furthermore, we can get an indication of the noise colour at higher frequencies from the trend in the CPMG measurements (Supplementary Fig. 7). From this analysis, we extract *β* ≈ 0.65 which represents the scaling exponent of a power-law noise spectrum, namely *S*_*ε*_(*f*) ∝ *f*^−*β*^. Comparing this value of *β* to the exponent of the measured low-frequency SHT charge noise spectrum, *β*_SHT_ = 1.33, we see that it differs significantly (Supplementary Fig. 2). This discrepancy has been observed in other hole spin qubit systems^10,17^, and indicates the noise spectrum is closer to a white noise spectrum (*β* = 0) at higher frequencies.

Qubit performance can be described more universally in terms of its fidelity, which evaluates the span of operations that are needed to perform two-axis control around the Bloch sphere. We evaluate the single qubit gate fidelity by performing standard randomised benchmarking (RB)^55,56^, which is insensitive to state preparation and measurement (SPAM) errors. This technique evaluates the fidelity of all gates from the Clifford group, which quantifies the rates of errors in the gate set.

To benchmark single-qubit gates, we generate the Clifford group using X/2 and Z/2 gates as primitives, with the Z/2 gate implemented as a virtual gate using phase updates of the microwave signal. The Clifford measurement versus the number of Clifford gates *m* is plotted in Fig. 5c, showing a decay in probability as the number of Clifford gates increases. We perform each Clifford gate sequence with even- and odd- parity measurement projections, and subtract the resulting probabilities from each other to obtain the difference *P*_even_( + Z) − *P*_even_( − Z) between the two final states. We fit the decay to the equation *c*_1_*f*^*m*^ + *c*_2_ from which we extract the Clifford fidelity *F*_C_ = (1 + *f*)/2. We find a single qubit fidelities of 99.83 ± 0.07% and 99.50 ± 0.19% for Q1 and Q2. These fidelities exceed the single qubit component of fault-tolerant threshold requirement of  ~ 99.4% ^23^ and are amongst the highest values achieved in silicon-based hole spin qubits^38^, and in natural silicon in general. The factor of  ≈ 3 difference in infidelities between Q1 and Q2 can likely be attributed to their differences in quality factor (factor of  ≈ 1.5), as well as differences in Rabi (factor of  ≈ 2) and dephasing time (factor of  ≈ 7).

For two-qubit gates, we estimate the physical upper limit to the fidelity from the two-qubit *Q*-factors. We evaluate the fidelity *F*_2Q_ from *F*_2Q_ = 3(1 + *f*)/4, where *f* is extracted from fitting the decay function *c*_1_*f*^*m*^ + *c*_2_ to the randomised benchmarking experiment^55^. From the extracted *Q*^CROT^ values, we obtain *F*_CROT_ ranging between 97% and 99%. More impressively, the estimated upper limit for *F*_DCZ_ is 99.7%, considerably higher than the fault-tolerance threshold^23^. This leaves ample scope for further techniques – such as substrate purification and advanced gate calibration – to achieve practical gate fidelities above the threshold, as measured through GST in ref. ^24^.

We emphasise that the key limitation in estimating the expected two-qubit fidelity from the exchange oscillation quality factor is that these oscillations probe only decoherence-induced errors. In practice, an unoptimized two-qubit benchmarking protocol introduces additional systematic error mechanisms, including pulse imperfections, qubit idling, crosstalk, and contextual errors^50^. However, these control-related errors can be substantially suppressed using advanced techniques such as pulse optimisation and active feedback. With such improvements, the measured two-qubit fidelities of the DCZ gate approach the values inferred from the exchange oscillation quality factor (see Supplementary Table II, where we show good agreement between the two methods for the DCZ gate). This agreement suggests that the exchange oscillation quality factor captures the intrinsic incoherent noise limit and thus serves as a useful indicator of the achievable two-qubit fidelity of the DCZ gate.

Notably, our coherence times and gate performance metrics could be improved by optimising the magnetic field direction and gate biases, as holes in silicon exhibit both highly anisotropic and voltage-sensitive qubit properties^7,17,27,57^. Additionally, we also measure the qubit relaxation time *T*_1_ = 255 ± 60 *μ*s (Supplementary Fig. 8), indicating that the dephasing time, rather than the relaxation time, is the limiting factor for these qubits. Follow-up studies could also explore the impact of gate-induced strain and device geometry on qubit properties, to provide insights into the variability of qubit performance as the system scales to larger numbers.

### Outlook

Our work demonstrates the feasibility of hole spin qubits in planar silicon MOS and adds a valuable new tool to the toolbox of silicon spin-based quantum computing. It verifies that hole spin qubits in planar silicon are directly compatible with their electron spin analogues and can even be operated in near-identical device structures with similar control schemes. Furthermore, hole spin qubits can operate with fast and high-fidelity quantum gates. They also offer low-power all-electrical control and strong spin-photon coupling^12^, providing key benefits to complement electron spin qubits that could be fabricated and operated on the same silicon CMOS chip. This work highlights the value of the development of devices using a 300 mm CMOS pilot line, which will facilitate the fabrication of large-scale SiMOS qubit architectures.

The fast and high-quality factor gates in natural silicon are surprising to witness, yet physically grounded – they provide a proof-of-principle result that fast and coherent operation can be achieved despite hyperfine coupling between hole spins and nuclear spins. As the hyperfine coupling is nonzero and tunable^16,58^, there is potential to further improve performance through isotopic purification and optimisation of quantum dot confinement and magnetic field orientation.

## Methods

The device is measured in a Bluefors LD250 dilution refrigerator with a base temperature of 10 mK and hole temperature of 130 mK. An external fixed magnetic field is supplied by an American Magnetics 6-3-1 magnet and points in the [110] direction of the silicon lattice. Low-frequency voltages are supplied with a Q-Devil QDAC-I, and the low-frequency lines pass through a Q-Devil 50 kHz cold filter. Fast voltage pulses are generated with a Quantum Machines OPX+ and are combined with low-frequency biases using an RC bias tee with a crossover frequency of 145 Hz. Microwave pulses are generated by a Rhode & Schwarz SGS100A Vector Signal Generator, using IQ upconversion. The in-phase and quadrature (I/Q) waveforms are generated by the OPX+. For the two-qubit control, a second SGS100A is used, and the two microwave pulses are combined using a power combiner. Microwave pulses are applied to the device via grapho-coax, with an additional 30 dB of in-line attenuation applied using discrete attenuators at different temperature stages within the fridge. Reflectometry is implemented by connecting the SHT ohmic to an external inductor *L* = 1200 nH, which forms a tank circuit with the parasitic capacitance *C*_p_. This creates an impedance match at a resonant frequency of *f*_0_ = 156 MHz. The reflectometry RF chain is configured with the incident signal delivered through 70 dB of in-line attenuation and a 12 dB directional coupler. The reflected signal is amplified at 4 K using a Cosmic Microwave CITLF2 low-noise amplifier, followed at 300K by two 20 dB ZFL-1000LN+ low-noise amplifiers. A diagram of the measurement setup is shown in Supplementary Fig. 1.

In this experiment, we use the (3,3)-(4,2) charge occupation for practical reasons. This charge transition was observed to have PSB and showed favorable tunneling rates for latched readout. Future studies could examine how qubit properties vary with charge occupation, particularly given the surprisingly weak *g*-factor variation in Si MOS devices found by^59^.

## Supplementary information

Supplementary Information

Transparent Peer Review file

## Data Availability

The data related to this study have been deposited in the Zenodo database, accessible at (https://doi.org/10.5281/zenodo.21065931)^60^.
