# Supplementary material for: Precision high-speed quantum logic with holes on a natural silicon foundry platform
Source: Nat Commun. 2026 Aug 27;17:10273. doi: 10.1038/s41467-026-76231-x (PMC13616933; doi:10.1038/s41467-026-76231-x)
Supplement: Supplementary file 1 [file 41467_2026_76231_MOESM1_ESM.pdf]

Precision high-speed quantum logic with holes on a natural silicon foundry platform

Isaac Vorreiter,[1] Jonathan Y. Huang,[1] Scott D. Liles,[1] Joe Hillier,[1] Ruoyu Li,[2] Bart Raes,[2] Stefan Kubicek,[2] Julien Jussot,[2] Sofie Beyne,[2] Clement Godfrin,[2] Sugandha Sharma,[2] Danny Wan,[2] Nard Dumoulin Stuyck,[3,4] Will Gilbert,[3,4] Chih Hwan Yang,[3,4] Andrew S. Dzurak,[3,4] Kristiaan De Greve,[2,5] and Alexander R. Hamilton[1]

[1]*School of Physics, University of New South Wales, Sydney, NSW 2052, Australia*
[2]*IMEC, Leuven, Belgium*
[3]*School of Electrical Engineering and Telecommunications, University of New South Wales, Sydney, NSW 2052, Australia*
[4]*Diraq Pty. Ltd., Sydney, NSW, Australia*
[5]*Department of Electrical Engineering (ESAT), KU Leuven, Leuven, Belgium*

SUPPLEMENTARY INFORMATION

CONTENTS

Supplementary information 1
S1. Experimental setup 2
S2. Single hole transistor charge noise 3
S3. Extended charge stability diagram 4
S4. Dot lever arms and hole temperature 5
S5. Extended single qubit Rabi oscillations 6
S6. Extended CROT oscillations 7
S7. Dynamical decoupling and noise analysis 8
S8. Spin relaxation time 9
S9. Clifford gates for single qubit randomised benchmarking 10
S10. Two-qubit fidelity estimate from quality factor 11

References 12

S1. Experimental setup

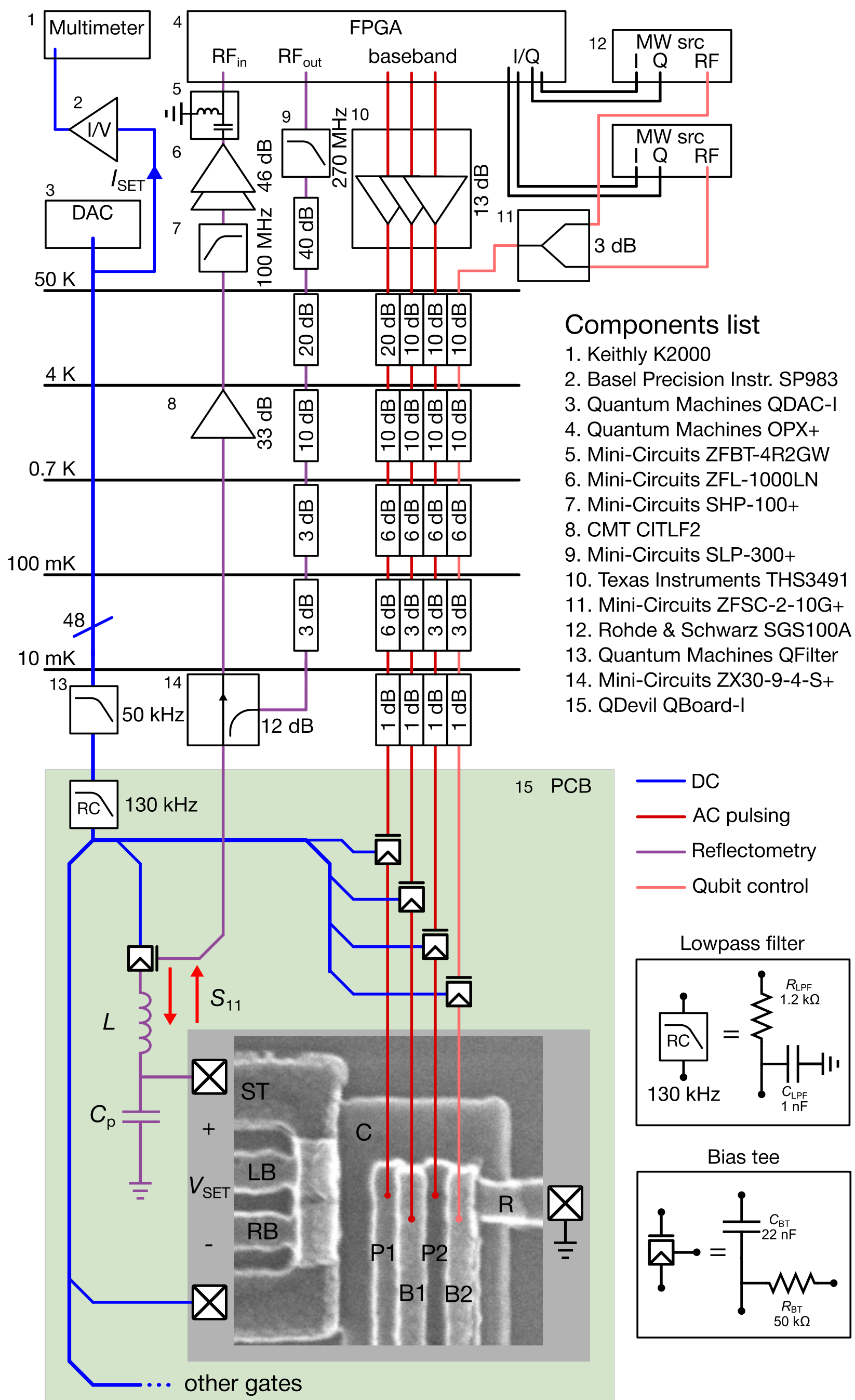

Supplementary Fig. 1 | Experimental setup.

S2. Single hole transistor charge noise

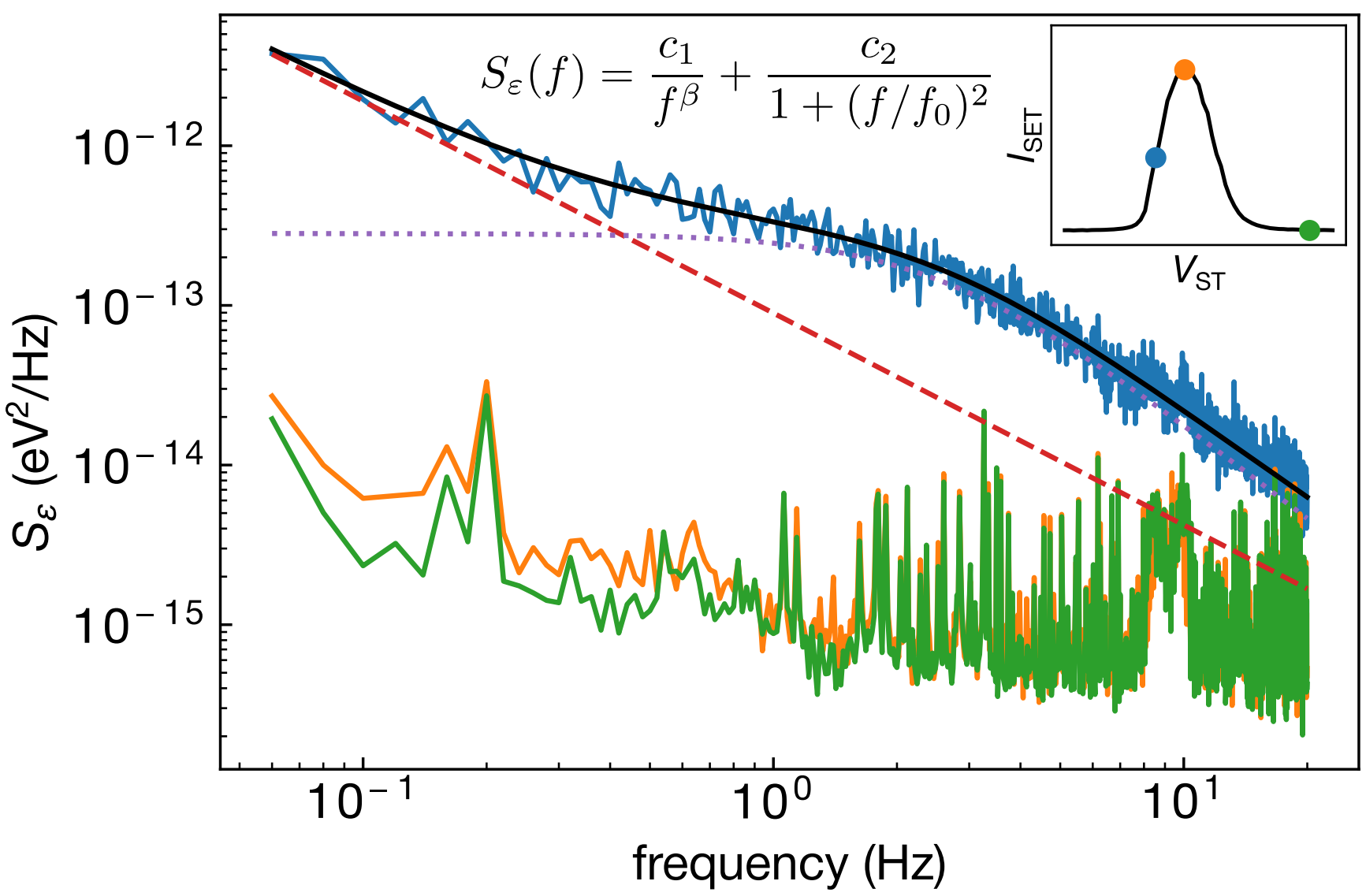

Supplementary Fig. 2 | Power spectral density of the charge noise of a single hole transistor. Charge noise power spectrum as measured through the single hole transistor. The current trace is converted to a charge power spectral density S_ε. Charge noise amplitude at 1 Hz is $\sqrt{S_\varepsilon} = 0.63\,\mu\text{eV Hz}^{-0.5}$. The spectrum follows a composite power-law c_1/f^β and Lorentzian $c_2/(1+(f/f_0)^2)$. From the fit (black), we extract a fit to the power law with an exponent of $\beta_{\text{SHT}} = \beta = 1.33 \pm 0.05$, and a fit to the Lorentzian with $f_0 = (2.6 \pm 0.2)\,\text{Hz}$. The individual power-law and Lorentzian fits are indicated by the dashed and dotted lines, respectively. Inset shows bias points on the SHT Coulomb oscillation where each spectrum was measured.

S3. Extended charge stability diagram

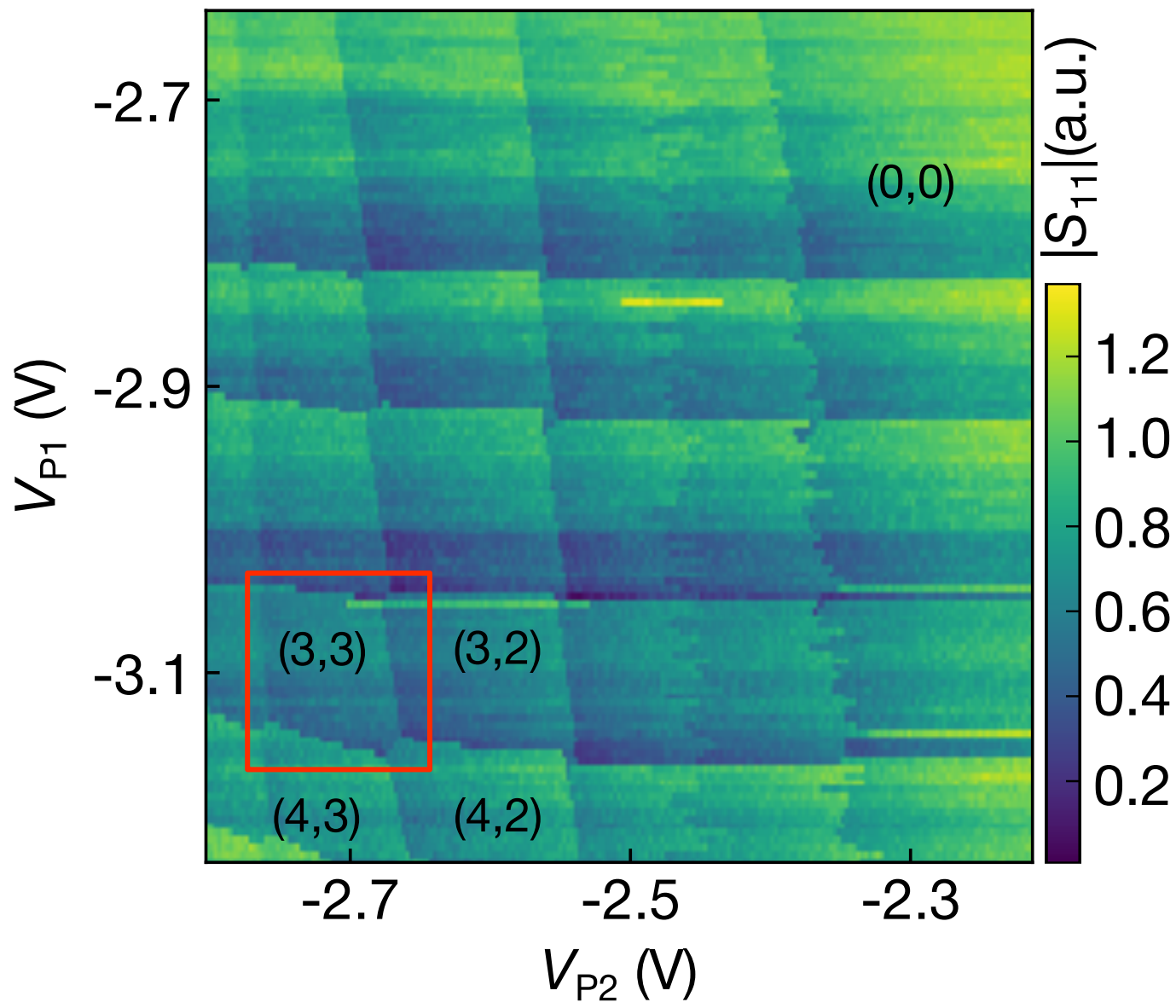

Supplementary Fig. 3 | Stability diagram showing control of the hole occupation in both dots down to the last hole. Full stability diagram of the device down to the last hole. The colour axis is given by $|S_{11}|$, which is proportional to the conductance through the SHT charge sensor. The (3,3)-(4,2) operating region depicted in the main text is indicated by the red square.

S4. Dot lever arms and hole temperature

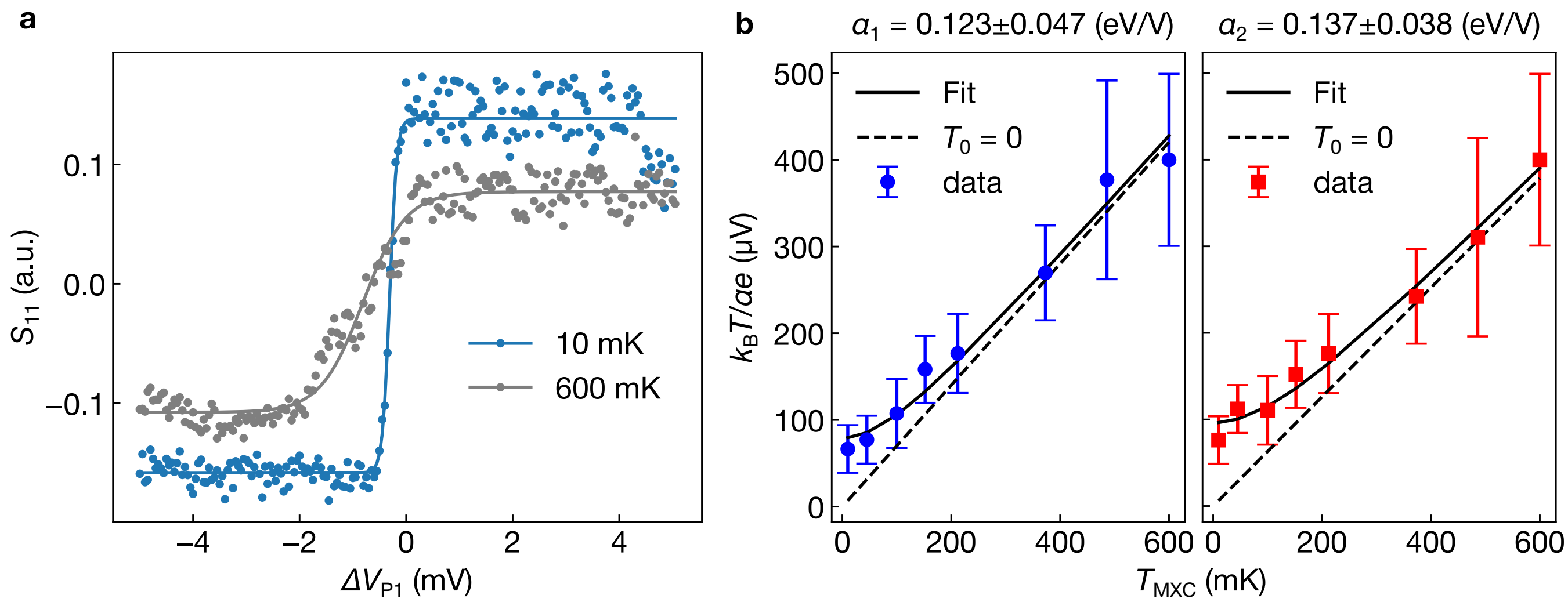

Supplementary Fig. 4 | Lever arm and hole temperature calculation. **(a)** SHT conductance measured by sweeping the P1 gate voltage V_{P1} at fridge temperatures of 15mK and 620mK. We repeat this measurement for the P2 gate voltage to extract both lever arms. **(b)** Plots of $k_BT/\alpha e$ resulting from the fits of the curves in (a) as a function of fridge mixing chamber temperatures T_{MXC}. From the fits we extract lever arms of $\alpha_1 = (0.123 \pm 0.047)\,\mathrm{eV\,V^{-1}}$ and $\alpha_2 = (0.137 \pm 0.038)\,\mathrm{eV\,V^{-1}}$ of the P1 and P2 plunger gates. The dotted lines show the inferred fit for the cases when $T_0 = 0$. Error bars are given from the 95% confidence interval to the fits of the datasets in (a).

In order to determine the charging energies, we need to be able to convert between the charging voltages (period of the stability diagram for V_{P1} and V_{P2}) to an energy. For quantum dot systems, this conversion factor is known as the lever arm α. Hence, we also need to ascertain the lever arms of P1 (α_1) and P2 (α_2) to their respective quantum dots in order to find the charging energy.

To find the lever arms, we measure the thermal broadening of the leads [1]. In Supplementary Fig. 4a we show the SHT reflectometry signal, S_{11}, for two different fridge mixing chamber temperatures T_{MXC}, measured across an $(N_1, N_2) \leftrightarrow (N_1+1, N_2)$ charge transition. The width of the transition is sensitive to the thermal broadening of the reservoir distribution of the hole states. We fit S_{11} to the function

$$S_{11} \propto \frac{1}{1+\exp\left(\frac{\Delta V_{\mathrm{P1}}\alpha_i e}{k_BT}\right)} = \frac{1}{1+\exp\left(\frac{\Delta V_{\mathrm{P1}}}{c_i}\right)} \tag{1}$$

where $\Delta V_{\mathrm{P1}} = V_{\mathrm{P1}} - V_0$, and V_0, $c_i = k_BT/\alpha_i e$ are fitting parameters. The smooth lines in Supplementary Fig. 4a show the fit to the raw data. We repeat these measurements for the $(N_1, N_2) \leftrightarrow (N_1, N_2+1)$ charge transition to extract the lever arm for P2 (not shown). From these fittings for P1 and P2, we plot $c_i = k_BT/\alpha_i e$ as a function of T_{MXC} in Supplementary Fig. 4b for both P1 and P2 measurements. We then fit this data to the function

$$y = \frac{k_BT}{\alpha_i e} = \frac{k_B}{\alpha_i e}\sqrt{T_{\mathrm{MXC}}^2 + T_0^2} \tag{2}$$

where α_i and T_0 are fitting parameters. Here, α_i represents the P_i gate lever arm, and T_0 represents the hole temperature. From the fits we extract lever arms of $\alpha_1 = (0.123 \pm 0.047)\,\mathrm{eV\,V^{-1}}$ and $\alpha_2 = (0.137 \pm 0.038)\,\mathrm{eV\,V^{-1}}$ of the P1 and P2 plunger gates. Additionally, we find an average hole temperature of $T_0 = (130 \pm 20)\,\mathrm{mK}$. The measured values of the lever arm reflect typical values in silicon MOS quantum dots (typically 0.05 to $0.2\,\mathrm{eV\,V^{-1}}$).

S5. Extended single qubit Rabi oscillations

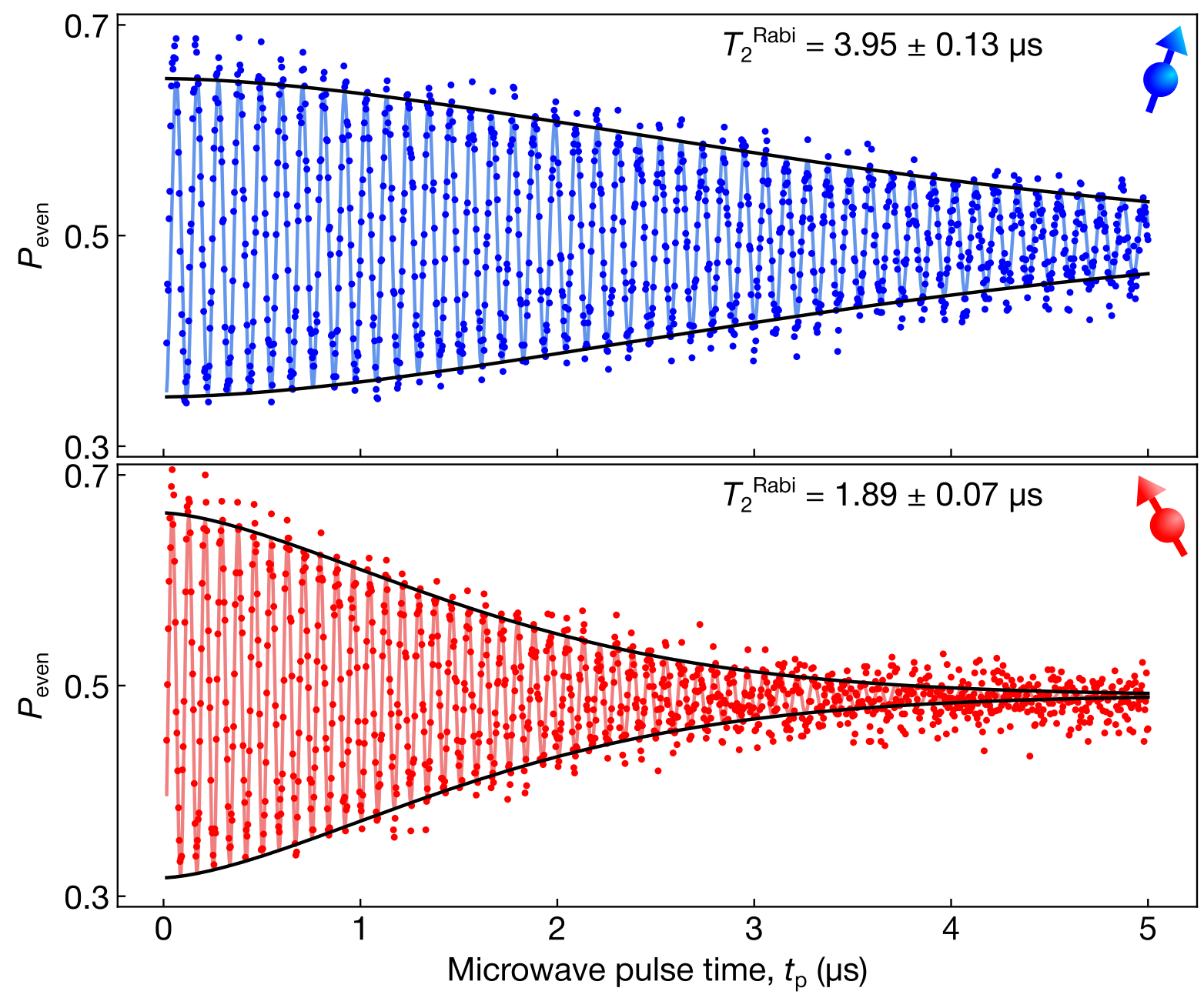

Supplementary Fig. 5 | Extended single qubit Rabi oscillations. Rabi oscillations for both Q1 and Q2 showing the decay of the oscillations past the $1/e$ point.

S6. Extended CROT oscillations

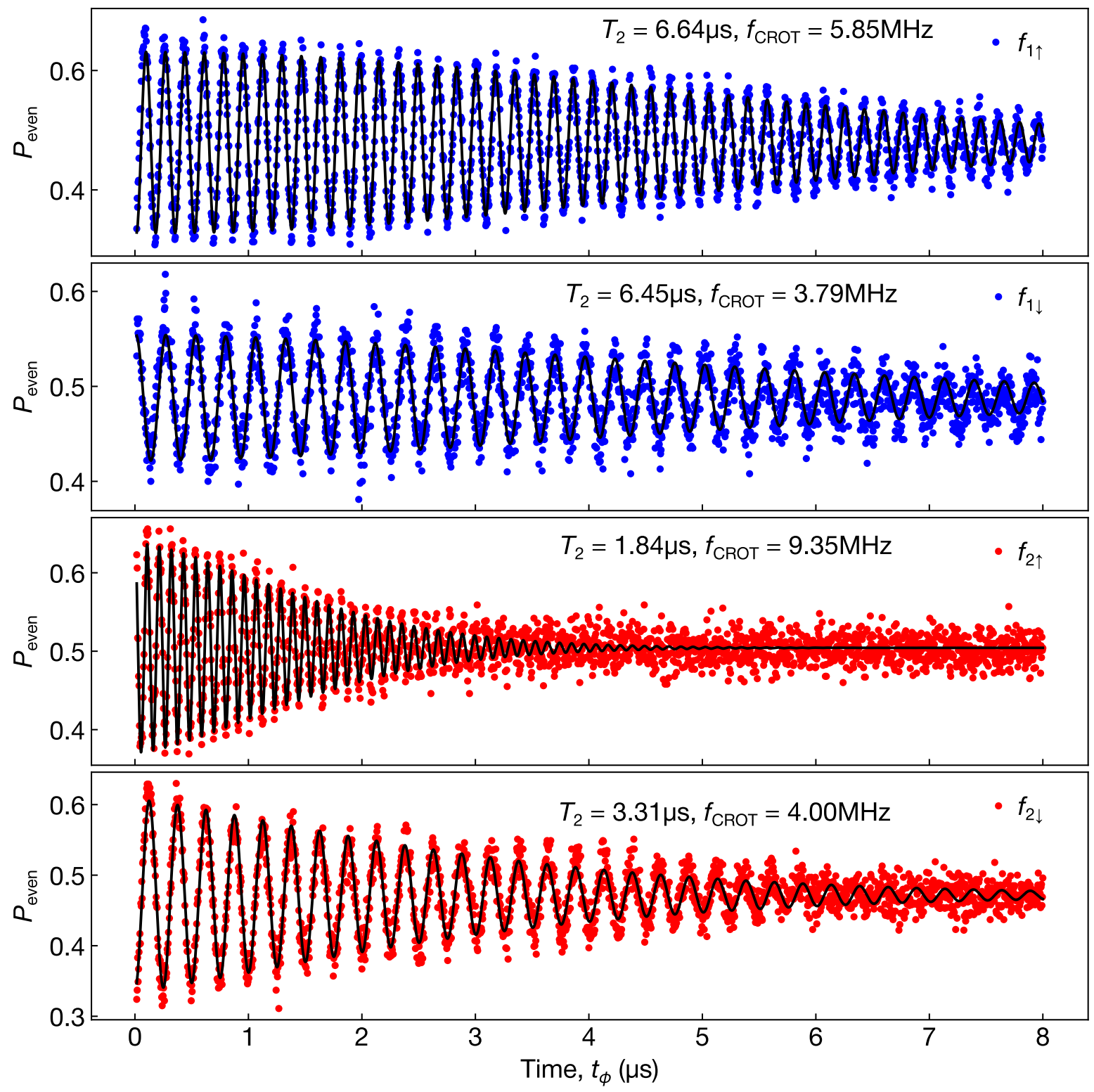

Supplementary Fig. 6 | Extended CROT oscillations. CROT oscillations for all four exchange-split EDSR branches with the control 'enabled'. Each trace is operated with the same J and using the same pulse amplitude.

S7. Dynamical decoupling and noise analysis

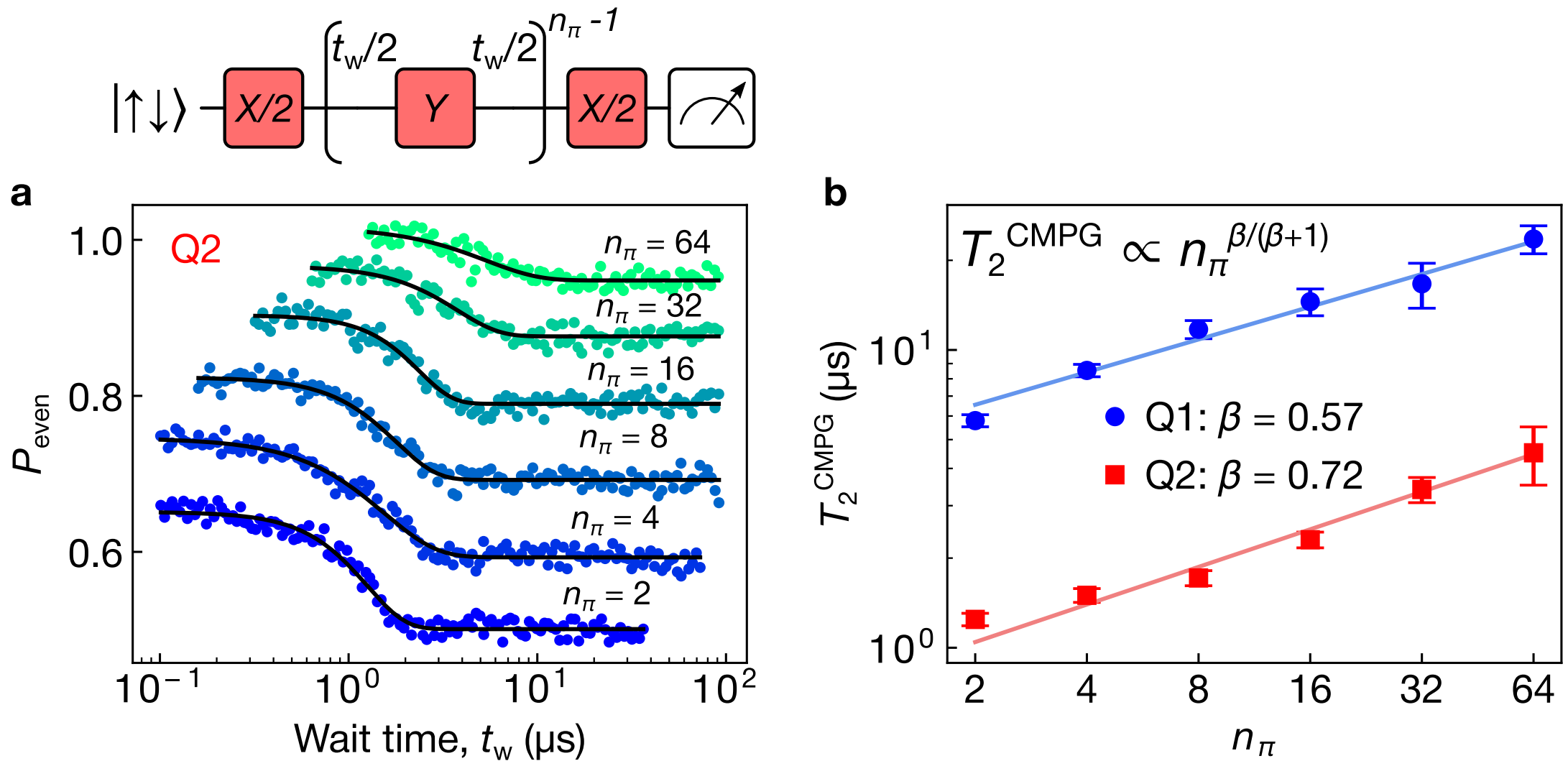

Supplementary Fig. 7 | CMPG and noise analysis. (a) CPMG dynamical decoupling decays for Q2. The total number of π-pulses, n_π, is indicated near the respective decay trace. The decays are fitted to $T_2^{\mathrm{CPMG}} = c_1 n_\pi^{\beta/(\beta+1)} + c_2$. **(b)** T_2^{CPMG} time versus total number of π-pulses n_π, yielding $\beta = 0.57 \pm 0.13$ and 0.72 ± 0.17 for Q1 and Q2 respectively. Error bars are given from the 95% confidence interval to the fits of the datasets in (a).

More generally, the echo experiment can be extended using the CPMG technique. By increasing the number of refocussing pulses, we expect an increase in the coherence times as the qubit is less exposed to 1/f noise. This is shown for Q2 in Supplementary Fig. 7a, where we plot the resulting decay for different n_π. In Supplementary Fig. 7b, we plot the fitted T_2^{CPMG} time as a function of the number of refocusing pulses n_π for Q1 and Q2. By fitting $T_2^{\mathrm{CPMG}} = c_1 n_\pi^{\beta/(\beta+1)}$, we extract β which represents the scaling exponent of a power-law noise spectrum, namely $S_\varepsilon(f) \propto f^{-\beta}$. These fits yield $\beta = 0.57 \pm 0.13$ and 0.72 ± 0.17 for Q1 and Q2 respectively. Furthermore, we compare these values of β from the Hahn and CPMG decays through the relation $\alpha = \beta + 1$. The decays yield an average of $\alpha = 1.68 \pm 0.41$ and 1.79 ± 0.29 for Q1 and Q2, which corroborate the values of β above.

S8. Spin relaxation time

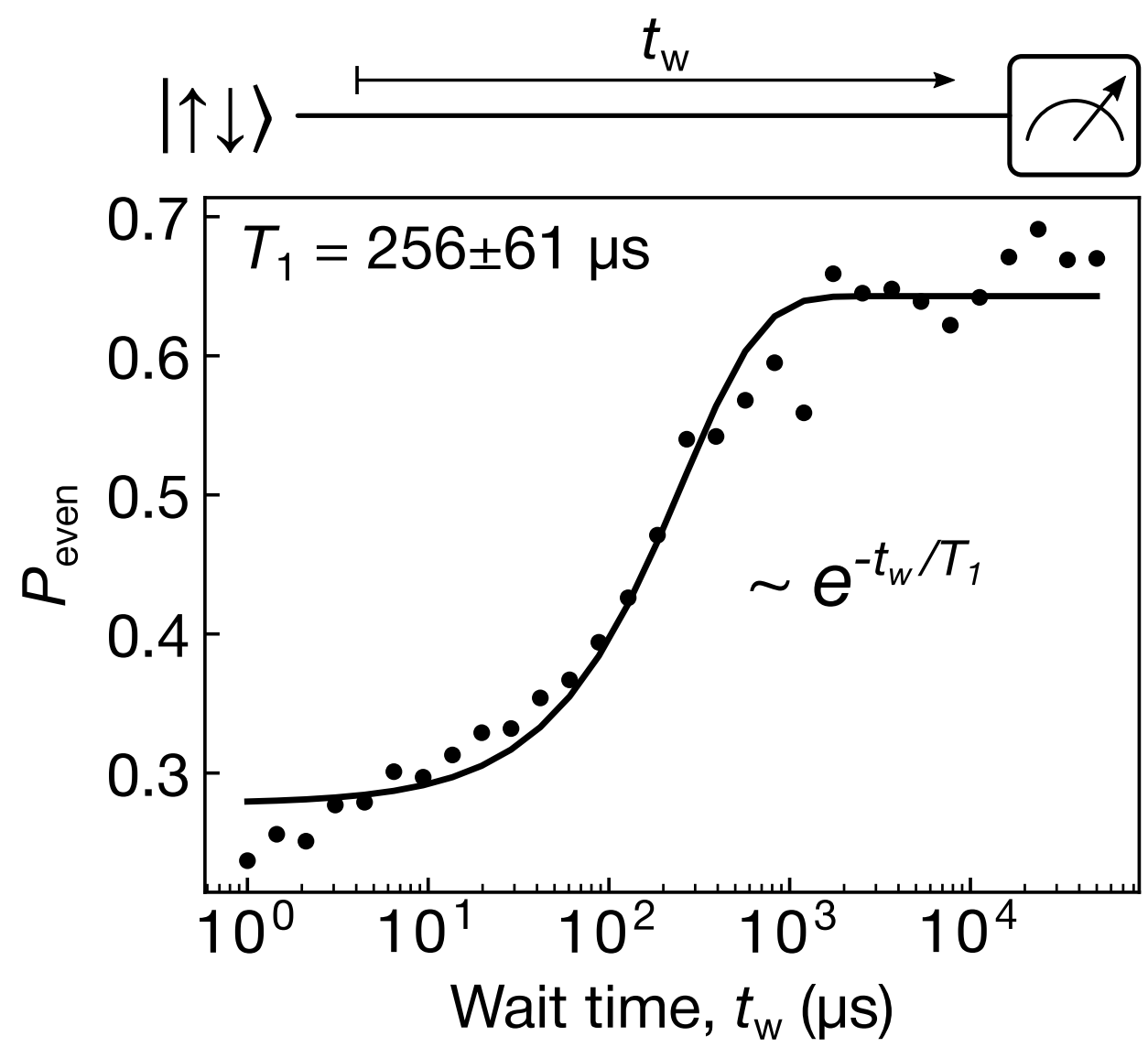

Supplementary Fig. 8 | Spin relaxation time. We probe the spin relaxation through a T_1 measurement. Here, we initialise the state into $|\uparrow\downarrow\rangle$ before waiting for time t_w before performing readout. During the wait, the state will inevitably relax into $|\downarrow\downarrow\rangle$. We plot the resulting spin probability as a function of t_w. After the spin relaxes, the resultant state maps to an even-parity outcome. The T_1 decay is described by the formula $c_1 \exp(-t_\mathrm{w}/T_1) + c_2$, from which we extract the spin relaxation time $T_1 = 255 \pm 61\,\mu\mathrm{s}$ measured at $B_\mathrm{ext} = 0.83\,\mathrm{T}$. This result compares similarly to the results measured in holes in silicon nanowires [2], and is on the same order of magnitude as recent theoretical predictions for this system [3]. Additionally, the T_1 time in Ge has been observed to have a strong dependence on the size of the lead tunnel rates [4]. To this end, we note that the lead tunnel rates in our device were not optimised for T_1 time. Broadly speaking, these results reflect a common trend for many spin qubit experiments, where $T_1 \gg T_2$ [5], meaning that the limiting factor for these devices is not the spin relaxation time.

S9. Clifford gates for single qubit randomised benchmarking

Clifford	X-Z decomposition
C_0	I
C_1	X
C_2	Z
C_3	Z, X
C_4	$X/2, Z/2$
C_5	$X/2, -Z/2$
C_6	$-X/2, Z/2$
C_7	$-X/2, -Z/2$
C_8	$Z/2, X/2$
C_9	$Z/2, -X/2$
C_{10}	$-Z/2, X/2$
C_{11}	$-Z/2, -X/2$
C_{12}	$X/2$
C_{13}	$-X/2$
C_{14}	$Z/2$
C_{15}	$-Z/2$
C_{16}	$-X/2, Z/2, X/2$
C_{17}	$-X/2, -Z/2, X/2$
C_{18}	$X, Z/2$
C_{19}	$X, -Z/2$
C_{20}	$Z, X/2$
C_{21}	$Z, -X/2$
C_{22}	$X/2, Z/2, X/2$
C_{23}	$-X/2, Z/2, -X/2$

Supplementary Table I. 24 single-qubit Clifford gate set constructed using elementary $\pi/2$ gates $\{X/2, X, Z/2, Z\}$. X and $X/2$ denote physical π and $\pi/2$ rotations about the x axis of the Bloch sphere, while Z and $Z/2$ denote virtual frame updates about the z axis. Each Clifford gates consists of one physical elementary gate on average, excluding the virtual Z and $Z/2$ gates.

S10. Two-qubit fidelity estimate from quality factor

We estimate the two-qubit gate fidelity from the quality factor of the exchange oscillations by relating the oscillation decay to the standard randomized benchmarking decay model. Assuming an exponential Clifford decay,

$$c_1 f^m + c_2 \sim e^{-m/Q}, \tag{3}$$

where f is the process polarisation, m is the number of Clifford gates and Q is the quality factor extracted from the exchange oscillations.

The process polarisation is related to the average gate fidelity F by [6]

$$f = 1 - \frac{d}{d-1}(1-F), \tag{4}$$

where $d = 2^n$ for a system of n qubits. For two qubits ($n = 2$), $d = 4$ and $F = F_{2Q}$.

Equating the two decay models and evaluating the decay after $m = Q$ Clifford operations gives

$$\left[1 - \frac{4}{3}(1 - F_{2Q})\right]^Q = e^{-1}. \tag{5}$$

Finally, solving for the two-qubit fidelity yields

$$F_{2Q} = \frac{1}{4} + \frac{3}{4}e^{-1/Q}. \tag{6}$$

We summarise literature values in Supplementary Table II for works in which both the two-qubit fidelity and the quality factor of the exchange oscillations are reported.

Ref.	Two-qubit quality factor	Maximum RB fidelity estimated from quality factor	Measured RB fidelity
[7]	~150	99.5%	99.15%
[8]	~150	99.5%	99.09%
[9]	~255	99.7%	99.65%

Supplementary Table II. Summary of two qubit fidelity measurements and comparison with the expected fidelity based on the quality factor of the entangling operation.

[1] Liles, S. D. *et al.* Spin and orbital structure of the first six holes in a silicon metal-oxide-semiconductor quantum dot. *Nature Communications* **9**, 3255 (2018).
[2] Piot, N. *A Qubit insensitive to noise*. Ph.D. thesis, Université Grenoble Alpes (2023).
[3] Wang, Z. *et al.* Electrical operation of hole spin qubits in planar mos silicon quantum dots. *Phys. Rev. B* **109**, 075427 (2024).
[4] Hendrickx, N. W., Franke, D. P., Sammak, A., Scappucci, G. & Veldhorst, M. Fast two-qubit logic with holes in germanium. *Nature* **577**, 487–491 (2020).
[5] Stano, P. & Loss, D. Review of performance metrics of spin qubits in gated semiconducting nanostructures. *Nature Reviews Physics* **4**, 672–688 (2022).
[6] Hashim, A. *et al.* Practical Introduction to Benchmarking and Characterization of Quantum Computers. *PRX Quantum* **6**, 030202 (2025).
[7] Huang, J. Y. *et al.* High-fidelity spin qubit operation and algorithmic initialization above 1 k. *Nature* **627**, 772–777 (2024).
[8] Steinacker, P. *et al.* Bell inequality violation in gate-defined quantum dots. *Nature Communications* **16**, 3606 (2025).
[9] Xue, X. *et al.* Quantum logic with spin qubits crossing the surface code threshold. *Nature* **601**, 343–347 (2022).
