# Supplementary material for: Precision high-speed quantum logic with holes on a natural silicon foundry platform
Source: Nat Commun. 2026 Aug 27;17:10273. doi: 10.1038/s41467-026-76231-x (PMC13616933; doi:10.1038/s41467-026-76231-x)
Supplement: Supplementary file 2 [file 41467_2026_76231_MOESM2_ESM.pdf]

nature portfolio

Peer Review File

Precision high-speed quantum logic with holes on a natural silicon foundry platform

Corresponding Author: Professor Alexander Hamilton

This manuscript has been previously reviewed at another journal. This document only contains information relating to versions considered at Nature Communications.

This file contains all reviewer reports in order by version, followed by all author rebuttals in order by version.

Version 0:

Reviewer comments:

Reviewer #1

(Remarks to the Author)
Vorreiter et al. replied extensively to the comments/issues of the 3 reviewers. With the correction made by the authors, the manuscript gained in clarity and misleading/vague statements have been removed. As a consequence the manuscript deserves publication in Nature Communications.

Reviewer #2

(Remarks to the Author)
The authors have addressed my questions and concerns. I find that the manuscript has improved considerably following the revisions, and I can now recommend publication in Nature Communications.

Reviewer #3

(Remarks to the Author)
In the manuscript "Precision high-speed quantum logic with holes on a natural silicon foundry platform" Vorreiter and authors demonstrated the universal operation of a hole in a silicon MOS device from an industrial foundry. They achieved record single qubit operation times of 99.4% and 99.8 percent in such a device. The authors carefully measured and analysed all error sources of the individual qubits. Q1 is limited by hyperfine noise while Q2 seems to be limited by charge noise.

I repeat that the results shown in the manuscript are impressive, very timely, and relevant for the community, following a global trend that semiconductor spin qubits are promising candidates for building a quantum processor.

The authors also addressed most of my points raised before. Despite not demonstrating full two-qubit logic, I recommend consideration in Nat. Comm. after addressing 3 remaining minor points.

p2, line 51: Please clarify whether this is the transversal effective mass.

p9, line 217: Please add that the estimation is based for a DCZ gate. Estimates for other two-qubit gates such as CROT are very different.
p9, line 218: The message in this sentence is too strong. I wouldn't call it a reliable predictor based on 3 data points. Noise correlations or the anisotropic exchange interaction can affect the estimations. Please either remove the sentence or weaken the statement.

Open Access This Peer Review File is licensed under a Creative Commons Attribution 4.0 International License, which permits use, sharing, adaptation, distribution and reproduction in any medium or format, as long as you give appropriate credit to the original author(s) and the source, provide a link to the Creative Commons license, and indicate if changes were made.
In cases where reviewers are anonymous, credit should be given to 'Anonymous Referee' and the source.
The images or other third party material in this Peer Review File are included in the article's Creative Commons license, unless indicated otherwise in a credit line to the material. If material is not included in the article's Creative Commons license and your intended use is not permitted by statutory regulation or exceeds the permitted use, you will need to obtain permission directly from the copyright holder.
To view a copy of this license, visit https://creativecommons.org/licenses/by/4.0/

Response to reviewer comments

We are grateful for the insightful feedback from all three reviewers, which has allowed us to improve the work significantly. We address below all questions raised by each reviewer and mention the corresponding changes made to the manuscript. Our responses are written in green, and the added text is in blue.

ADDED COMMENTS

REVIEWERS' COMMENTS

Reviewer #1 (Remarks to the Author):

Vorreiter et al. replied extensively to the comments/issues of the 3 reviewers. With the correction made by the authors, the manuscript gained in clarity and misleading/vague statements have been removed. As a consequence the manuscript deserves publication in Nature Communications.

Reviewer #2 (Remarks to the Author):

The authors have addressed my questions and concerns. I find that the manuscript has improved considerably following the revisions, and I can now recommend publication in Nature Communications.

Reviewer #3 (Remarks to the Author):

In the manuscript "Precision high-speed quantum logic with holes on a natural silicon foundry platform" Vorreiter and authors demonstrated the universal operation of a hole in a silicon MOS device from an industrial foundry. They achieved record single qubit operation times of 99.4% and 99.8 percent in such a device. The authors carefully measured and analysed all error sources of the individual qubits. Q1 is limited by hyperfine noise while Q2 seems to be limited by charge noise.

I repeat that the results shown in the manuscript are impressive, very timely, and relevant for the community, following a global trend that semiconductor spin qubits are promising candidates for building a quantum processor.

The authors also addressed most of my points raised before. Despite not demonstrating full two-qubit logic, I recommend consideration in Nat. Comm. after addressing 3 remaining minor points.

1. p2, line 51: Please clarify whether this is the transversal effective mass.

We thank the reviewer for this helpful suggestion. We have clarified this in the manuscript. The revised text now reads:

[Section 1, paragraph 4]:
"The in-plane effective mass of the holes is approximately $0.6m_0$-$0.7m_0$ [29], and the ground state is expected to be primarily heavy-hole-like [27]."

p9, line 217: Please add that the estimation is based for a DCZ gate. Estimates for other two-qubit gates such as CROT are very different.

We thank the reviewer for this point of clarification. We have clarified in the manuscript that the discussion refers specifically to the DCZ gate. The revised text now reads:

[Section 4, paragraph 6]:
"With such improvements, the measured two-qubit fidelities of the DCZ gate approach the values inferred from the exchange oscillation quality factor (see Supplementary S10 where we show good agreement between the two methods for the DCZ gate)."

p9, line 218: The message in this sentence is too strong. I wouldn't call it a reliable predictor based on 3 data points. Noise correlations or the anisotropic exchange interaction can affect the estimations. Please either remove the sentence or weaken the statement.

We thank the reviewer for this helpful suggestion. We agree that the exchange oscillation quality factor should not be interpreted as a definitive predictor of two-qubit fidelity, since additional effects such as noise correlations and anisotropic exchange interactions can influence gate performance. Our intention was to emphasize that it provides an estimate of the intrinsic incoherent noise limit, and thus an upper bound on the achievable fidelity. We have therefore softened the wording in the manuscript to reflect this more clearly. The revised text now reads:

[Section 4, paragraph 6]:
"This agreement suggests that the exchange oscillation quality factor captures the intrinsic incoherent noise limit and thus serves as a useful indicator of the achievable two-qubit fidelity of the DCZ gate."

Errata

In addition to changes requested by the referees we have corrected and updated the manuscript as follows

1) Clarified definition of error bars in Figure 2, subpanel (d):

[Figure 2, caption]:
“The Rabi frequencies are extracted using the same fitting function as in (a), with error bars representing the 95% confidence intervals of the fits.”

2) Clarified definition of error bars in Supplementary Figure 4, subpanel (b):

[Supplementary Figure 4, caption]:
“Error bars are given from the 95% confidence interval to the fits of the datasets in (a).”

3) Clarified definition of error bars in Supplementary Figure 7, subpanel (b):

[Supplementary Figure 7, caption]:
“Error bars are given from the 95% confidence interval to the fits of the datasets in (a).”
